# Automation and artificial intelligence in radiation therapy treatment planning

**DOI:** 10.1002/jmrs.729

**Published:** 2023-10-04

**Authors:** Scott Jones, Kenton Thompson, Brian Porter, Meegan Shepherd, Daniel Sapkaroski, Alexandra Grimshaw, Catriona Hargrave

**Affiliations:** ^1^ Radiation Oncology Princess Alexandra Hospital Raymond Terrace Brisbane Queensland Australia; ^2^ Department of Radiation Therapy Services Peter MacCullum Cancer Care Centre Melbourne Victoria Australia; ^3^ Northern Sydney Cancer Centre Royal North Shore Hospital Sydney New South Wales Australia; ^4^ Monash University Clayton Victoria Australia; ^5^ RMIT University Melbourne Victoria Australia; ^6^ W.P Holman Clinic Royal Hobart Hospital Hobart Tasmania Australia; ^7^ Queensland University of Technology, Faculty of Health, School of Clinical Sciences Brisbane Queensland Australia

## Abstract

Automation and artificial intelligence (AI) is already possible for many radiation therapy planning and treatment processes with the aim of improving workflows and increasing efficiency in radiation oncology departments. Currently, AI technology is advancing at an exponential rate, as are its applications in radiation oncology. This commentary highlights the way AI has begun to impact radiation therapy treatment planning and looks ahead to potential future developments in this space. Historically, radiation therapist's (RT's) role has evolved alongside the adoption of new technology. In Australia, RTs have key clinical roles in both planning and treatment delivery and have been integral in the implementation of automated solutions for both areas. They will need to continue to be informed, to adapt and to transform with AI technologies implemented into clinical practice in radiation oncology departments. RTs will play an important role in how AI‐based automation is implemented into practice in Australia, ensuring its application can truly enable personalised and higher‐quality treatment for patients. To inform and optimise utilisation of AI, research should not only focus on clinical outcomes but also AI's impact on professional roles, responsibilities and service delivery. Increased efficiencies in the radiation therapy workflow and workforce need to maintain safe improvements in practice and should not come at the cost of creativity, innovation, oversight and safety.

## Graphical abstract

This commentary highlights the way AI has begun to impact radiation therapy treatment planning and looks ahead to potential future developments in this space. Radiation therapists will play an important role in how AI‐based automation is implemented into practice in Australia, ensuring its application can truly enable personalised and higher‐quality treatment for patients.

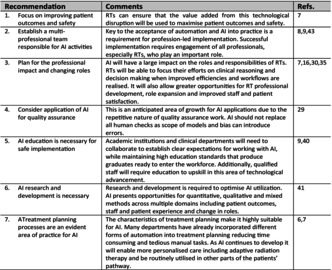

## Background

The Royal Australian and New Zealand College of Radiology (RANZCR) ‘State of Play 2019’ paper on artificial intelligence (AI) states that the field of radiation oncology has always been at the forefront of technology adoption in the healthcare industry.^1^ For many radiation oncology departments, the adoption of AI to improve their radiation therapy planning processes has already begun, albeit to varying degrees and with a range of methodological approaches. It is expected that AI and the automated systems it enables will be the next large‐scale technological change to radiation therapy. Throughout the healthcare literature,^2^ use of the term AI has become synonymous with many forms of automation as well as machine learning prediction to reduce repetitive menial tasks and expedite clinical decision‐making. It is important to acknowledge that while forms of automation have long been engineered into software systems used in the clinical setting, this commentary focuses on the contemporary practice of AI‐driven automation systems underpinned by machine learning methods, and therefore, we use the terms AI and automation interchangeably. While full‐scale AI that can perform high‐order cognitive decision‐making is yet to be achieved in this space, this field of research will continue to expand and impact radiation therapy beyond current capabilities of treatment planning software.^2^ As it does so, radiation therapists (RTs) in Australia, whose education, training and responsibilities include both treatment and treatment planning, are in an advantageous position to ensure that the value added from this technological disruption will be used to maximise patient outcomes and safety. In this commentary, we highlight the way AI has begun to impact radiation therapy treatment planning and look ahead to potential future developments in this space, both clinically and professionally.

It is clear that the automated and predictive abilities of contemporary software that provide advances in plan quality and enhanced treatment planning workflow will change the role of RTs, locally and globally, in the future.^3^, ^4^ Australian RTs will need to work closely with our colleagues in radiation oncology and medical physics,^1^ and be positioned to lead the safe implementation of AI. Furthermore, the application of AI has the potential to reduce time and inter‐observer variability and increase accuracy in the delivery of radiation therapy.^5^ The characteristics of treatment planning make it highly suitable for automation. Incorporating machine learning and automated functions in the treatment plan development process will improve access to personalised care and adaptive radiation therapy techniques which will allow RTs, who are responsible for treatment planning, to focus their efforts on clinical reasoning and decision‐making.^6^

Emerging technologies provide new opportunities to improve patient care and create value like the transformative and disruptive technologies of the past have done.^7^ The key shift for RTs will be to have clinical input and guidance at every stage in order to use it to its greatest advantage. For this to occur RT‐driven research will need to be a priority to foster knowledge, experience and credibility in this area. Key to the acceptance of automation and AI into practice is a requirement for profession‐led implementation with an emphasis on safety and accuracy,^8^ with RTs, radiation oncologists (ROs) and radiation oncology medical physicists (ROMPs) supported to adapt alongside the technology.^9^

This paper describes the anticipated implications of these methods to Australian radiation therapists when applied to the various aspects of dosimetric treatment plan development. An executive summary of the key points can be found in Table 1.

**Table 1:** Executive summary of commentary key points.

Recommendation	Comments	References
Focus on improving patient outcomes and safety	RTs can ensure that the value added from this technological disruption will be used to maximise patient outcomes and safety	[7]
2 Establish a multi‐professional team responsible for AI activities	Key to the acceptance of automation and AI into practice is a requirement for profession‐led implementation. Successful implementation requires engagement of all professionals, especially RTs, who play an important role	[8, 9, 43]
3 Plan for the professional impact and changing roles	AI will have a large impact on the roles and responsibilities of RTs. RTs will be able to focus their efforts on clinical reasoning and decision‐making when improved efficiencies and workflows are realised. It will also allow greater opportunities for RT professional development, role expansion and improved staff and patient satisfaction	[7, 16, 30, 35]
4 Consider application of AI for quality assurance	This is an anticipated area of growth for AI applications due to the repetitive nature of quality assurance work. AI should not replace all human checks as scope of models and bias can introduce errors	[29]
5 AI education is necessary for safe implementation	Academic institutions and clinical departments will need to collaborate to establish clear expectations for working with AI, while maintaining high education standards that produce graduates ready to enter the workforce. Additionally, qualified staff will require education to upskill in this area of technological advancement	[9, 40]
6 AI research and development is necessary	Research and development is required to optimise AI utilisation. AI presents opportunities for quantitative, qualitative and mixed methods across multiple domains including patient outcomes, staff and patient experience and change in roles	[41]
7 Treatment planning processes are an evident area of practice for AI	The characteristics of treatment planning make it highly suitable for AI. Many departments have already incorporated different forms of automation into treatment planning reducing time‐consuming and tedious manual tasks. As AI continues to develop, it will enable more personalised care including adaptive radiation therapy and be routinely utilised in other parts of the patients' pathway	[6, 7]

AI, artificial intelligence; RT, radiation therapist.

## Automated Image Segmentation (Auto‐Contouring)

**Figure 1:**
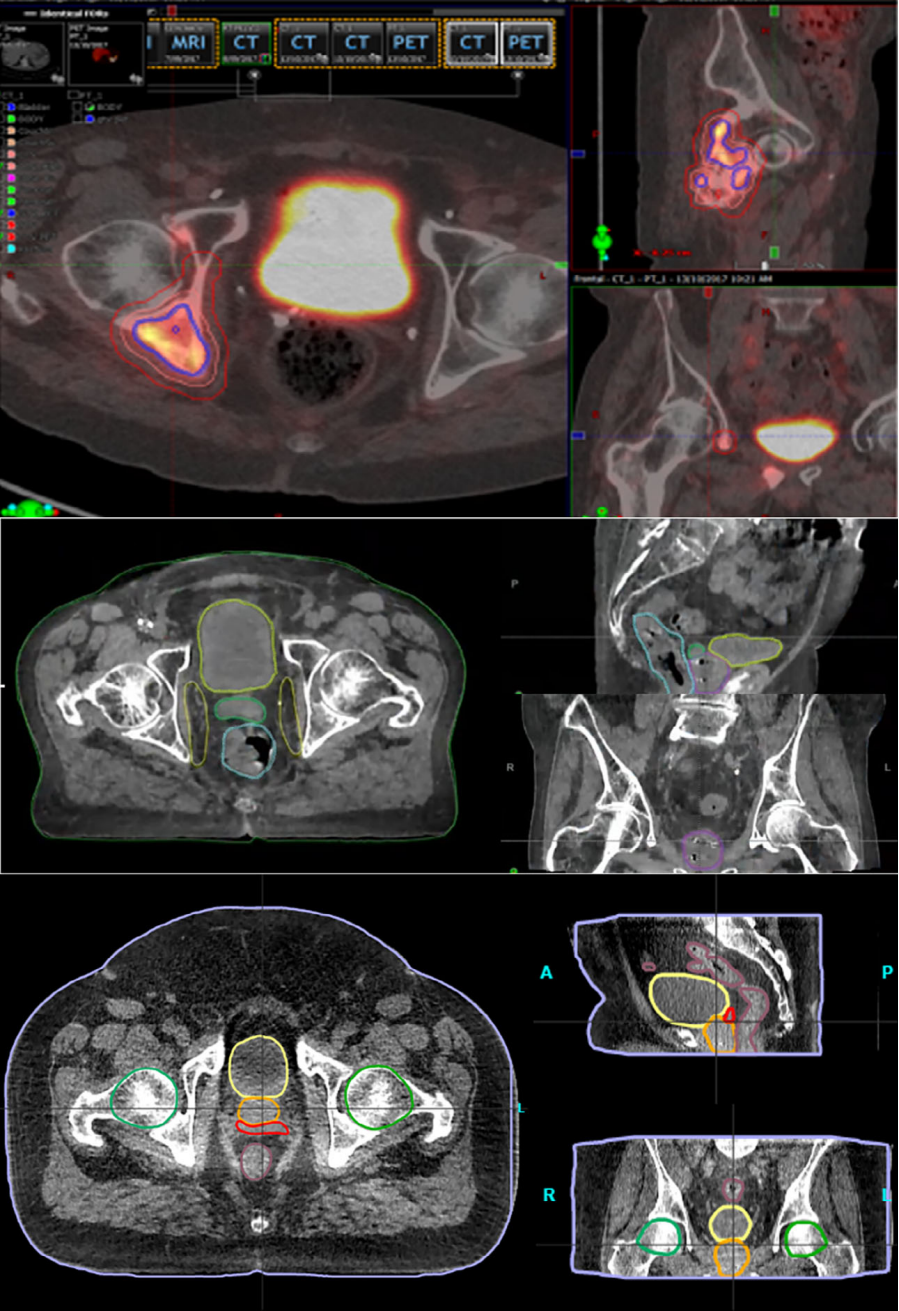
Auto‐segmentation precision examples (A, B, C) on PET‐CT, CT and CBCT guided contouring accuracy for pelvis organs at risk and targets using three current vendor software products.

Manual image segmentation is a time‐consuming task^10^, ^11^ routinely performed in radiation therapy to identify each patient's targets and organs at risks (OAR) and can be subject to significant inter‐ and intra‐observer variability. The efficacy and safety of treatment planning requires accurate segmentation for optimisation and to assess plan quality. Auto‐segmentation techniques have been clustered into three generations of algorithms, with multi‐atlas‐based and hybrid techniques being considered the state‐of‐the‐art.^11^, ^12^ More recently, however, the accelerated development and early application of deep learning (DL) algorithms for auto‐segmentation suggests we are entering the next generation.^13^ Figure 1 demonstrates auto‐segmentation results using current vendor clinical software. Widespread application of these techniques will require a collaborative effort with our radiation oncology colleagues to ensure the contours developed to meet their requirements for accurate treatment and dose reporting.^14^ Beyond the standard requirements for OAR delineation, it is expected that the use of this technology for target delineation will become more prevalent and integral to streamline workflows.^15^ This will rely on clinician oversight to ‘approve’ the contours and provide feedback to vendors on model performance,^16^ with an increased use of multimodal imaging. Where RTs are generating target volume contours using AI tools, there are scope of practice and appropriate training implications.^17^ There will also be products emerging that check contours inside QA software and this may be important for online adaptive radiation therapy. Institutions need to document their efforts with contouring accuracy studies, or at least store them for later analysis. This provides the opportunity to research not only the clinical impacts of this technology but also the impact to professional roles, responsibilities and service delivery.

## Dose Optimisation

High‐quality brachytherapy and external beam radiation therapy (EBRT) treatments are underpinned by the dosimetric plan developed for the individual patient. Development of more personalised plans has become a feasible option through the widespread application of modulated EBRT techniques such as intensity modulated radiation therapy (IMRT) and volumetric modulated arc therapy (VMAT). As the complexity of planning has increased, so have the efforts in finding solutions to reduce associated increased planning times.

One of the first available options for reducing planning time was user‐defined scripts and templates, applied in treatment planning systems. Both scripts and templates allowed repetitive tasks like beam arrangement and objective creation to be automated based on departmental‐defined protocols. This enabled more time to be spent on the optimisation and refinement of plans rather than menial tasks common to most plans. More recent developments in automated planning have included techniques such as multi‐criteria optimisation^2^, ^18^ amongst others to expedite the iterative process of DVH objective selection and manipulation to generate personalised treatment plans for each individual patient.^19^, ^20^

The next phase of dosimetry automation is likely to use more complex machine learning algorithms combined with voxel‐based data to determine the optimal dosimetric outcome for each individual based on spatial data.^21^, ^22^, ^23^ This will allow predictions to move beyond DVH outcomes and describe endpoints such as conformity and gradient measurements as well as optimised tumour control probability (TCP) and normal tissue complication probability (NTCP) linked to individualised outcomes.^21^ Further beyond this is the potential to use biological functional responses to radiation treatment to guide adaptive treatments.^24^ RTs currently contribute to most parts of the plan development process; however, as the scope of the AI algorithms increases this may subsequently require an adjustment to the RT role. Ultimately AI development may lead to the ability to feed patient parameters from diagnosis and initial consultation, in symphony with local templates, to produce a radiation therapy treatment plan (or plans), and the assessment of suitability can also be automated based on objective criteria.

**Figure 2:**
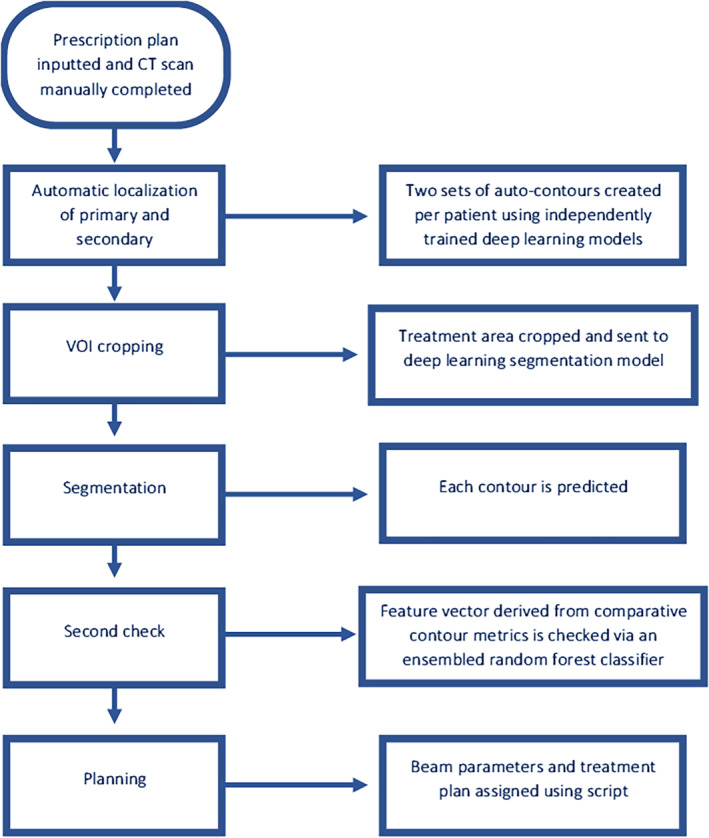
Example workflow of AI automated palliative dosimetric plan development as described by Netherton et al.^23^

Vendors will soon introduce ‘software‐as‐a‐service’ models,^25^ meaning patient data are uploaded to a vendor's cloud‐based service before a radiation therapy plan is sent back. It is likely as development in this area continues plan assessment skills will remain but ultimately all elements of treatment plans, for common cases, may be fully automated. Ethical and legal implications of AI performing all tasks, with access to sensitive data, are yet to be completely resolved.^26^ Some organisations may opt for an in‐house approach to meet their specific needs. A recent example from Netherton et al.^27^ focused on an automated treatment planning framework for spinal radiation therapy. Their AI framework was developed as an automated vertebral contouring second check to decrease chances of mislabelling of vertebrae and associated time pressures around emergency cases. Figure 2 demonstrates how their AI framework completes all steps of generating an acceptable treatment plan.

## Quality Assurance

Quality assurance (QA) of radiation therapy treatment plans comprises of many facets including a review of the dosimetric aspects of the plan or imaging dataset. In addition to the dosimetric aspects, there are many parameters to review to ensure that the plan is clinically deliverable and meets the departmental policies and guidelines. Since treatment plan evaluation requires the repetitive checking of similar plan parameters for each patient, this process is ideal for automation and may be more effective for quality, safety and efficiency than policies and procedures alone.^7^

Utilisation of scripting has been shown to reduce the amount of time spent reviewing plan parameters that must be individually validated for each treatment (e.g. calculation model, field dose rate) and enable RTs to have more time for evaluating dosimetric plan quality.^28^ It is anticipated that this area will continue to evolve to become a more streamlined process in the treatment pathway as checks that are currently scripted become incorporated into vendor software and AI‐based methods increase.^29^

## Professional Impact

Implementation of radiation therapy planning automation solutions is expected to have a large impact on the roles and responsibilities of planning dosimetry both for local and international RTs in the near future.^30^ Already, in some departments, AI implementation has presented opportunities to capitalise on efficiencies and redeploy planning staff to other tasks, such as treatment or research roles.^16^ This is likely to be the case for many departments as they see a decrease in planning times, requiring less dosimetry headcount to manage the workload. A recent published survey has shown that RTs generally feel optimistic about the application of AI in radiation therapy and the impact AI will have on their role.^31^ It would be remiss to acknowledge that not all RTs will share this viewpoint and that the full benefits of these systems may not be recognised until they are a mature part of the clinical ecosystem. This requires careful planning and well‐executed implementation strategies to maximise engagement and collaboration between all key stakeholders. It is important that this collaboration extends to our patients. Consumer engagement forms part of the Australian National Safety and Quality Health Service Standards as outlined in Standard 2 Partnering with Consumers.^32^ A recent survey in the UK found there was moderately negative patient view towards the use of AI in radiotherapy, demonstrating the importance of engaging with patients in Australia as AI is adopted into local practice.^33^

It is further recognised that implementation of new technology brings other responsibilities in ensuring automation applied is safe and effective, not the least of which is the education of current staff and students.^34^, ^35^ With reduced dosimetry staff headcount with greater planning throughput, we must consider potential concerns surrounding professional burnout.^36^ However, application of AI technology could hold promise to be a productive force,^7^ significantly improving planning efficiency and workflow, reducing time‐consuming and tedious manual tasks, lightening the mental load and promoting greater opportunities for RT professional development, role expansion and staff and patient satisfaction.^37^

Furthermore, the increased uptake of adaptive planning methods on the treatment machines presents opportunities for staff to expand their skillset or even extend their current scope of practice. This may include, but not limited to, advanced image interpretation skills, advanced radiological anatomy knowledge^38^ and leveraging of planning knowledge and expertise to credentialing for adaptive planning on the treatment machine.^39^ This could potentially result in hybrid roles where treatment RTs are required to employ their planning skills to assess the accuracy of segmentation and ensure the specified treatment intent is met for online adaptive plans on a daily basis. Widespread application of such roles across Australia will require published evidence‐based practice regarding what dose variation thresholds should be used clinically when considering daily online adaption due to position and shape change.^40^, ^41^, ^42^

## Education

Education of students and academic programmes are also expected to be impacted by this change. Clinical placements typically incorporate allocated time for dosimetry skill development. In addition to foundational dosimetry principles, an understanding of the radiation therapy planning automation modelling process, potentially including foundations in data science and machine learning, may also be a reasonable expectation for students and academic institutions given the importance of understanding the assumptions underpinning these methods. Support for this has not only been described in the literature,^43^ but has become a part of the professional standards set out by the Medical Radiation Practice Board of Australia in their statement on AI in medical radiation practice.^44^ Adoption of these professional capabilities will require both academic institutions and clinical departments to collaborate on the expectations for student training and the responsibilities held by both parties so clear expectations can be set, while maintaining high education standards that produce graduates ready to enter the workforce.^9^

## Research

Research into AI in healthcare has exponentially grown across a broad spectrum of topics since being introduced as a technology that can perform specific tasks better or as well as humans. This includes domains such as image analysis in radiology derived from machine learning or deep learning AI. Research on AI in RT has focused on standardising care and promoting efficiency in RT work practices, through safety and efficacy studies of AI technologies, applied across various body sites. New and novel AI research employs mixed methodologies to investigate domains such as the relationship between patient outcomes and use of AI, big data radiomics for planning precision and personalisation,^45^ comparisons of AI technologies in RT and assessing the qualitative impact on relinquishing traditional RT planning roles to AI technology. Radiation therapist‐led and inter‐professional research into AI in radiation therapy will be central to future RT role expansion as in time, computing power and accuracy of AI technologies will replace traditional RT tasks. Further research into the application of AI in radiation therapy is imperative for ensuring our profession adapts appropriately. In particular, studies with a focus on staffing models, education and the health economic impact of this technology are prime areas requiring robust research work.

## Considerations

The implementation of AI in radiation therapy treatment planning raises concerns at the reduction of human intervention in radiation therapy processes and the potential reduction of innovation and development through curiosity. It is important that there is an understanding of how current AI‐based methods solve clinical problems to ensure the safe support of high‐quality care.

Without knowledge of how this new technology works and the appropriate quality assurance required, it can be potentially seen as a ‘Black Box’ method where outcomes are unable to be verified as logical and plausible.^7^, ^26^ As such, RTs will need to assess and recognise when automated methods and predictive models perform well and act when they do not. Risk assessment and quality assurance of AI technologies to identify sub‐optimal solutions will require RTs to develop and maintain appropriate skills related to model interpretability, explainability and data dependency.^8^ This may be particularly challenging as new RTs enter the workforce after automated approaches are implemented.

Successful implementation requires human engagement of all tripartite professionals,^46^ especially RTs, who play an important role at the core of treatment planning. Implemented in the right way, AI has the potential to substantially speed up the process, reduce the time burden of human intervention, especially for repetitive tasks, and improve timely access to high‐quality personalised radiation therapy while maintaining the balance between promotion of patient wellbeing and minimising harm.^47^

## Conclusion

The prospect of widespread implementation of AI‐based automation and decision support in Australian radiation oncology departments presents both challenges and opportunities for radiation therapists. Formal discussions between the multidisciplinary groups are ongoing and seen to be critically important in the process of redefining roles and expectations of each professional group. While a gap currently exists between AI developments and widespread uptake, this means there is still space in which to prepare the RT workforce and to examine the possible implications on clinical practice, patient care and cancer care delivery. A key challenge in the increased use of AI in radiation therapy is to ensure there is still room for human intervention, development (invention) and research in the face of increasing complexity of problems and solutions. Increased efficiencies in the radiation therapy workflow and workforce need to maintain safe improvements in practice and should not come at the cost of creativity, innovation, oversight and safety within the profession.

## Conflict of Interest

The authors declare no conflict of interest.

## Data Availability

Data sharing is not applicable to this article as no new data were created or analyzed in this study.
